# Novel screening system revealed that intracellular cholesterol trafficking can be a good target for colon cancer prevention

**DOI:** 10.1038/s41598-019-42363-y

**Published:** 2019-04-17

**Authors:** Shingo Miyamoto, Takumi Narita, Masami Komiya, Gen Fujii, Takahiro Hamoya, Ruri Nakanishi, Shuya Tamura, Yurie Kurokawa, Maiko Takahashi, Michihiro Mutoh

**Affiliations:** 1 Division of Prevention, Center for Public Health Sciences, National Cancer Center, Tokyo, Japan; 2 Central Radioisotope Division, National Cancer Center Research Institute, Tokyo, Japan; 3 Present Address: Department of Cancer Cell Research, Sasaki Institute, Sasaki Foundation, Tokyo, Japan

**Keywords:** Cancer prevention, Cancer prevention

## Abstract

In conventional research methods for cancer prevention, cell proliferation and apoptosis have been intensively targeted rather than the protection of normal or benign tumor cells from malignant transformation. In this study, we aimed to identify candidate colon cancer chemopreventive drugs based on the transcriptional activities of TCF/LEF, NF-κB and NRF2, that play important roles in the process of malignant transformation. We screened a “validated library” consisting of 1280 approved drugs to identify hit compounds that decreased TCF/LEF and NF-κB transcriptional activity and increased NRF2 transcriptional activity. Based on the evaluation of these 3 transcriptional activities, 8 compounds were identified as candidate chemopreventive drugs for colorectal cancer. One of those, itraconazole, is a clinically used anti-fungal drug and was examined in the Min mouse model of familial adenomatous polyposis. Treatment with itraconazole significantly suppressed intestinal polyp formation and the effects of itraconazole on transcriptional activities may be exerted partly through inhibition of intracellular cholesterol trafficking. This screen represents one of the first attempts to identify chemopreventive agents using integrated criteria consisting of the inhibition of TCF/LEF, NF-κB and induction of NRF2 transcriptional activity.

## Introduction

Colorectal cancer (CRC) is the third most common cancer worldwide. Nearly 1.4 million new cases occurred in 2012, which is expected to increase to 2.4 million cases worldwide annually by 2035^1^. Despite extensive efforts to develop anti-cancer drugs over the past century, the pace of drug development lags behind the increasing rate of CRC mortality. Therefore, additional approaches to control the development of this cancer are in great demand^2^. In this context, primary prevention, including chemoprevention, is an important therapeutic strategy. We believe that prevention by means of “drug re-positioning” is the best strategy to fight this malignancy in terms of low toxic side effects and costs. Certain drugs, such as FDA-approved agents, are safer than unknown natural compounds, and their assumed modes of action could support a clinical trial. Another advantage of this strategy is that the number of drugs that require evaluation is limited. Thus, this strategy may eliminate the need to extensively search for chemopreventive cancer agents, as has been done previously.

Cancer chemoprevention refers to the use of natural, synthetic or biological agents to reverse, suppress or prevent either the initial phases of carcinogenesis or the progression of premalignant cells to invasive disease^3^. Hanahan D and Weinberg RA^4^ previously demonstrated that cell proliferation and apoptosis are important early hallmarks of cancer. Therefore, cell proliferation and apoptosis have been intensively targeted in the conventional screening of cancer preventive drugs. In addition, another primary target in cancer prevention is the protection of normal or benign tumor cells from malignant transformation, not the killing of cancer cells. However, less research has been conducted targeting the initiation and promotion stages of colon carcinogenesis. Therefore, to determine novel cancer preventive drug candidates, new targets specific to prevention might be required. In this study, we focused on the three statuses, differentiation, inflammation and oxidative stress, which are involved in the initiation and promotion stages of colon carcinogenesis.

Most colon cancers are associated with mutations in *APC* or *CTNNB1*, and ~90% of colon cancers are associated with defects in the canonical Wnt signaling pathway^5^. *APC* or *CTNNB1* mutations cause nuclear translocation, and the TCF/LEF-binding of β-catenin protein results in the up regulation of *c-Myc* and *LGR5* expression^6,7^. Clevers *et al*. reported that TCF/LEF activity is crucial for maintaining intestinal stem cells in crypt homeostasis under physiological conditions, and aberrant activation of Wnt signaling has a central function in the carcinogenic process via promotion of proliferating and maintaining the undifferentiated status of cells^8^. Because *APC* restoration showed rapid tumor regression by promoting cellular differentiation and reestablishing crypt homeostasis in established tumors *in vivo*^9^, suppression of TCF/LEF activity is presumed to be an appropriate target for cancer prevention.

The relative risk of CRC in patients with inflammatory bowel diseases, including Crohn’s disease (CD) and ulcerative colitis (UC), has been estimated to be between 4- to 20-fold^10^. In this context, chronic inflammation has been considered to play a major role in CRC initiation, promotion and progression. Nuclear factor-κB (NF-κB)^11^ is a common transcription factor that regulates several inflammation-related genes, such as *cyclooxygenase-2* (*COX-2*)^12^, *inducible nitric oxide synthase* (*iNOS*)^13^, *interferon-γ* (*IFN-γ*), *tumor necrosis factor-α* (*TNF-α*) and *interleukin-1β* (*IL-1β*)^14^. These genes are overexpressed in inflamed mucosa and colonic neoplasms. The aberrant activation of NF-κB is reported in over 50% of CRCs^15^, and the genetic deletion of IKKβ, which activates NF-κB in epithelial cells, dramatically reduced tumor incidence in a mouse model of colitis-associated cancer^16^. This evidence suggests that there is a direct implication of NF-κB signaling in the early stages of colon carcinogenesis.

On the other hand, chronic activation of NF-κB induces the promotion of epithelial cell turnover and the generation of reactive oxygen species (ROS)^17^. Induction of DNA mutations by ROS attack appears to be principally involved in the early stage of colon carcinogenesis, which is linked to inflammatory processes^18^. ROS could thereby overwhelm the tissue’s antioxidant defenses and induce DNA damage. One of the major players in the antioxidant defense system of various tissues is nuclear factor-erythroid 2-related factor 2 (NRF2)^19^. NRF2 is a basic leucine zipper redox-sensitive transcriptional factor that plays a central role in the regulation of antioxidant and/or detoxifying genes. Interestingly, NRF2-knockout mice showed increased susceptibility to dextran sulfate sodium (DSS)-induced inflammation in the colorectum (colitis)^20^ and carcinogenesis^21^ compared with wild-type mice.

Thus, inflammation and oxidative stress play important roles in the initiation and promotion stages of colon carcinogenesis. Therefore, drugs that inhibit the maintenance of the undifferentiated status and suppress inflammation as well as diminish oxidative stress could be good candidate chemopreventive agents for CRC.

In this study, we established a luciferase-based cell assay system driven by TCF/LEF, NF-κB and NRF2 transcriptional factors, which play major roles in the signaling that maintains the undifferentiated state, inflammation and anti-oxidative stress, respectively. Drugs in the validated library were screened and evaluated with integrated criteria for the 3 types of transcriptional activity. Of note, the combined evaluation of the activity of the 3 transcriptional factors is one of the novel points of our system.

## Results

### Establishment of cell-based TCF/LEF-, NF-κB- and NRF2-luciferase reporter screening system

**Figure 1:**
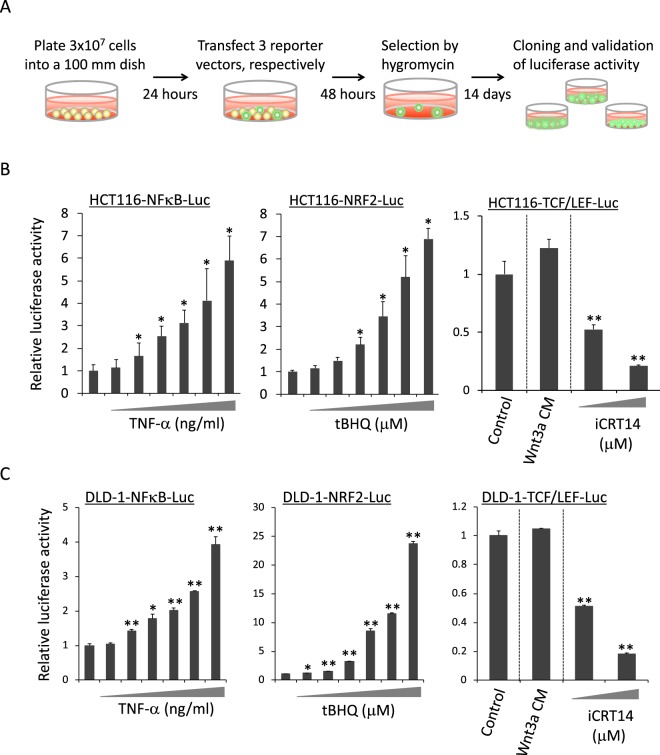
Design and validation of a high-throughput screening system based on the transcriptional activities of TCF/LEF, NF-κB and NRF2. (**A**) Workflow for the establishment of each cell lines for the screening system. The respective inducers or inhibitors were added for 24 hours to validate the sensitivity of the luciferase assay in each reporter cell lines of HCT116 (**B**) and DLD-1 (**C**). Luciferase activity was measured after stimulation with 0, 1.25, 2.5, 5, 10, 20 and 40 ng/ml TNF-α in NF-κB-reporter cell lines. Luciferase activity was measured after stimulation with 0, 1.25, 2.5, 5, 10, 20 and 40 μM tBHQ in NRF2-reporter cell lines. TCF/LEF-reporter cell lines were stimulated by Wnt3a CM and 5 and 10 μM iCRT14 in HCT116 cells or 20 and 40 μM iCRT14 in DLD-1 cells. Wnt3a CM was added at 10% of total volume. The data are shown as the relative value, with the value of the vehicle-treated cells set as 1 in each cell line. The data are the mean ± SD, n = 3. *p < 0.03, **p < 0.003 *vs*. control (vehicle-treated cells). CM, conditioned medium.

Since ~90% of colon cancers are associated with defects in the canonical Wnt signaling pathway, we selected colon cancer cell lines HCT116 and DLD-1 with mutations in *APC* and *CTNNB1*, respectively. As an initial step, HCT116 and DLD-1 cell lines were transfected with TCF/LEF-, NF-κB- and NRF2-reporter vectors to developed stable transfectants (Fig. 1A). After transfection, hygromycin selection and cloning, each clone was evaluated for sensitivity to the activators of NF-κB (TNF-α) and NRF2 (tBHQ). Because the HCT116 and DLD-1 cell lines have mutated β-catenin and APC, a clone was selected for screening based on having high luciferase activity in the basal state. After evaluation with the above criterion, each reporter cell line used in screening was selected out of 27, 11, 9, 28, 32 and 9 clones, and named HCT116-TCF/LEF-Luc, HCT116-NFκB-Luc, HCT116-NRF2-Luc, DLD-1-TCF/LEF-Luc, DLD-1-NFκB-Luc and DLD-1-NRF2-Luc, respectively (data not shown). The selected clones were validated by their sensitivities to the NF-κB activator, NRF2 activator and TCF inhibitor (iCRT14), respectively. Each clone showed a clear, dose-dependent increase in luciferase activity by TNF-α or tBHQ stimulation and reduction of luciferase activity by iCRT14 (Fig. 1B,C).

### Screening of validated library based on three types of transcriptional activities

**Figure 2:**
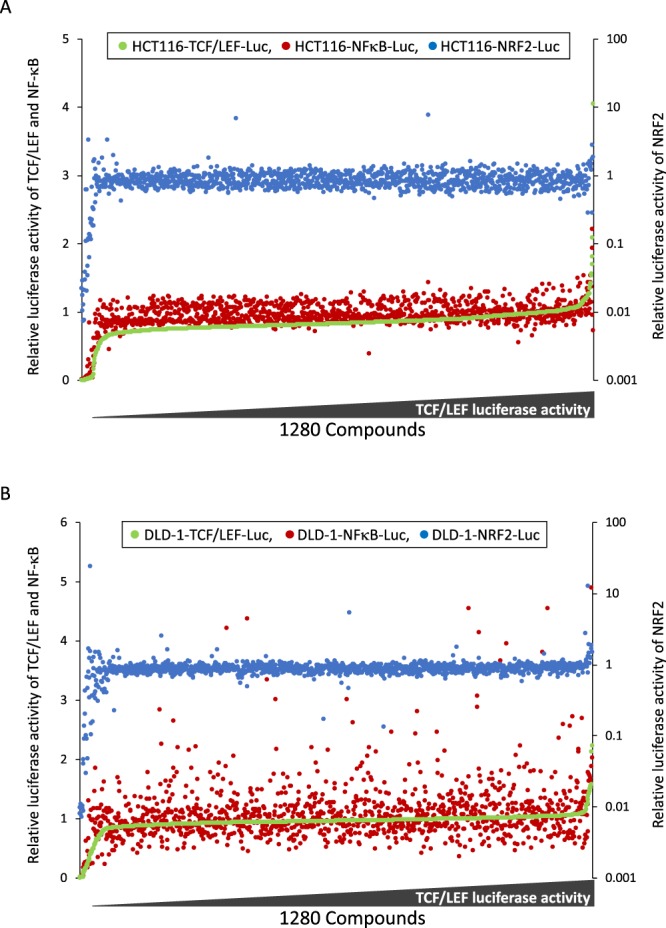
Primary screening of validated library based on the decrease of TCF/LEF- and NF-κB-luciferase activity and increase of NRF2-luciferase activity. Relative luciferase activity was evaluated 24 hours after adding each compound in the validated library at 1 μM in HCT116 (**A**) and DLD-1 (**B**) reporter cell lines. Relative luciferase activities of TCF/LEF, NF-κB and NRF2 in the cells treated with each compound (X-axis) are plotted from ascending TCF/LEF activity (Y-axis). The data are shown as relative values, with the value from vehicle-treated cells set as 1 in each cell lines.

To explore drugs that decrease TCF/LEF- and NF-κB-dependent transcriptional activities and increase NRF2-dependent transcriptional activity, we screened the validated library (Open Innovation Center for Drug Discovery, The University of Tokyo). The validated library is composed of 1280 drugs approved by the FDA. The threshold for the selected compounds was set as less than 0.8 for TCF/LEF- and NF-κB-dependent transcriptional activities and more than 1.2 for NRF2-dependent transcriptional activity, which represents the relative ratio compared to the control value. With these thresholds, 490 and 61 drugs decreased the TCF/LEF-dependent transcriptional activity in HCT116 (Fig. 2A) and DLD-1 (Fig. 2B) cells, respectively. In terms of the NF-κB-dependent transcriptional activity, 95 and 273 drugs decreased it in HCT116 and DLD-1 cells, respectively. Meanwhile, NRF2-dependent transcriptional activity was induced by 59 and 24 drugs in HCT116 and DLD-1 cells, respectively. Although many drugs decreased or increased the individual transcriptional activities, only 8 drugs (alexidine dihydrochloride, benzethonium chloride, bortezomib, docetaxel, exemestane, fenbendazole, ITZ, pyrvinium pamoate) fulfilled the combined criterion of suppressing TCF/LEF- and NF-κB- and inducing NRF2-dependent transcriptional activity (Table 1).

**Table 1:** Hit compounds selected by the first screening.

Drugs	Original uses	Relative luciferase activity			Cell lines
		TCF/LEF	NF-κB	NRF2	
Alexidine dihydrochloride	Antibacterial	0.16	0.62	1.60	HCT116
Benzethonium chloride	Antibacterial	0.51	0.65	1.53	HCT116
Bortezomib	Antineoplastic	0.40	0.22	23.87	DLD1
Docetaxel	Antineoplastic	0.38	0.41	1.52	DLD1
Exemestane	Antineoplastic	0.65	0.78	3.28	HCT116
Fenbendazole	Anti-intestinal parasites	0.59	0.77	1.46	DLD1
Itraconazole	Antifungal	0.53	0.70	1.21	HCT116
Pyrvinium pamoate	Anthelmintic	0.02	0.30	3.34	HCT116

Relative luminescence unit of DMSO control was set as 1.0 in each cell lines.

### Dose-dependent effects of identified drugs on transcriptional activity

**Figure 3:**
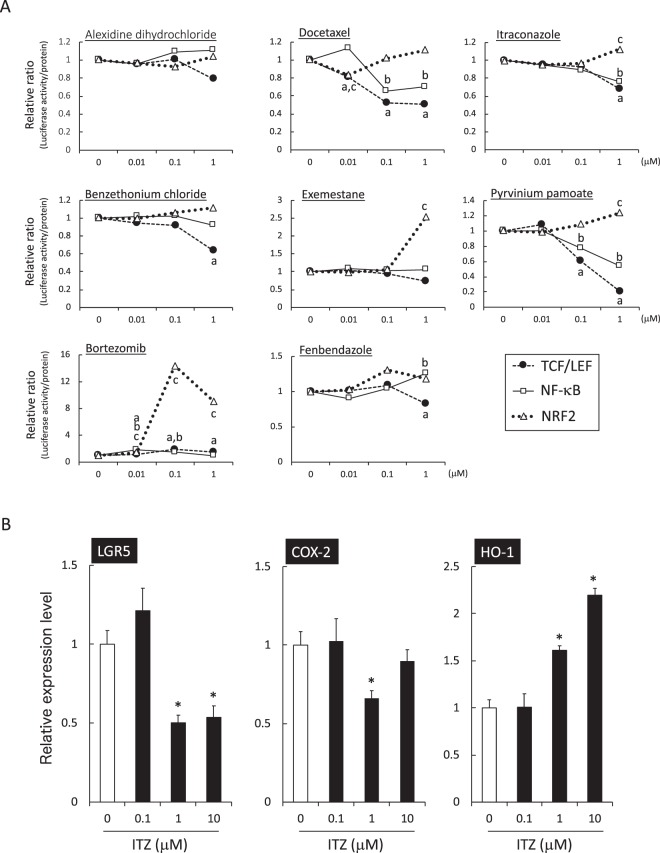
Secondary screening of primary hits with the data of a strong candidate, itraconazole. (**A**) The effects of the 8 compounds that were primary hits by luciferase activity were evaluated and normalized by protein concentration. The dose-dependent effects of each compound on luciferase activity were evaluated. The data are indicated as the mean, n = 3. ^a^*p* < 0.05 *vs*. control in TCF/LEF. ^b^*p* < 0.05 *vs*. control in NF-κB. ^c^*p* < 0.05 *vs*. control in NRF2. (**B**) The effects of a strong candidate, ITZ, on the expression levels of *LGR5*, *COX-2* and *HO-1* mRNA, which are target genes of TCF/LEF, NF-κB and NRF2, respectively, were evaluated by RT-qPCR in HCT116 parental cells. HCT116 parental cells were cultured in medium containing the indicated dose of ITZ for 6 hours. The data were normalized to GAPDH expression. Each expression level in the control (0 μM ITZ) was set as 1. The data are the mean ± SD, n = 3. *p < 0.05 *vs*. control (0 μM ITZ).

Primary hits from the first screening may contain false-positive results due to the toxic effects caused by the apparent suppression of luciferase activity. To eliminate false-positive hits and validate the first screening results, we further analyzed the dose-dependent effects of 8 identified drugs on the transcriptional activity followed by the protein assays in HCT116 and DLD-1 cell lines, respectively. Consistent with the first screening, benthethonium chloride, docetaxel, exemestane, ITZ and pyrvinium pamoate significantly suppressed the TCF/LEF-dependent transcriptional activity at 1 μM (Fig. 3A). NF-κB-dependent transcriptional activity was decreased by docetaxel, ITZ and pyrvinium pamoate. Meanwhile, the NRF2-dependent transcriptional activity was induced by benthethonium chloride, bortezomib, exemestane, ITZ and pyrvinium pamoate. In the second screening, only two drugs, ITZ and pyrvinium pamoate, fulfilled our combined criteria. We further evaluated the effects of hit candidates on Wnt, NF-κB and NRF2 signaling pathways in another cell line, HT29, and the results are shown in Fig. S1. In this experiment, we obtained similar results, especially with ITZ (10 µM) and pyrvinium pamoate (1 µM) treatment, to those we observed in the HCT116 cells. Consistent with our findings, Li *et al*. previously reported pyrvinium as a chemotherapeutic agent for intestinal polyposis^22^. Therefore, we focused on ITZ as a novel candidate for a colon cancer chemopreventive agent and applied it to the next experiments.

To validate the effects of ITZ on practical gene expression, *LGR5*, *COX-2* and *HO-1*, which represent the target genes of TCF/LEF, NF-κB and NRF2, were evaluated in the HCT116 parental cell line. As shown in Fig. 3B, ITZ induced a reduction of *LGR5* and *COX-2* expression levels, which was in contrast to the induction of *HO-1* expression.

### Suppression of intestinal polyp formation in Min mice by ITZ administration

**Figure 4:**
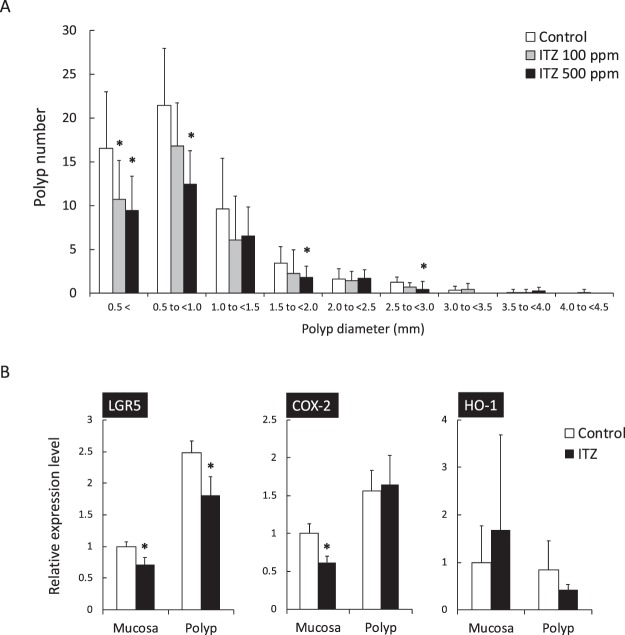
The effects of ITZ on the size distribution of intestinal polyps and on the expression of TCF/LEF, NF-κB and NRF2 target genes in Min mice. (**A**) Min mice were fed a basal diet (open box), 100 ppm (gray-filled box) and 500 ppm (black-filled box) of an ITZ-containing diet for 8 weeks. The number of polyps per mouse in each class is given as the mean ± SD. *p < 0.05 *vs*. 0 ppm. (**B**) Quantitative real-time PCR analysis was performed to determine *LGR5*, *COX-2* and *HO-1* mRNA expression levels in the non-polyps or polyps of Min mice with or without 100 ppm ITZ for 8 weeks. The data are normalized to *GAPDH* expression. Each expression level in the non-polyps of the untreated group was set as 1. The data are the mean ± SD, n = 4. *p < 0.05 *vs*. 0 ppm.

For further investigation, the effect of ITZ treatment on intestinal polyp formation was evaluated using Min mice, *Apc*-mutant mice that are models of familial adenomatous polyposis (FAP). Min mice were administered 100 or 500 ppm ITZ for 8 weeks starting from 5-weeks of age. ITZ administration did not affect body weight, food intake or clinical symptoms, such as appearance of the hair coat and activity throughout the experimental period. In addition, no changes were observed in the weights and the macroscopic view of major organs, including the liver, spleen and kidney that may have been attributable to toxicity (data not shown). As shown in Table 2, the total number of intestinal polyps was significantly decreased to 71% and 60% in the 100 and 500 ppm-treated group compared with the untreated control value, respectively. Notably, 500 ppm of ITZ treatment significantly suppressed polyp formation in the entire small intestine. In terms of polyp diameter, ITZ especially suppressed polyp formation to a smaller size (Fig. 4A).

**Table 2:** Number of intestinal polyps/mouse in Min mice treated with or without ITZ.

Dose (ppm)	No.	Total	Small intestine			Colon
			Proximal	Middle	Distal	
0	10	54.1 ± 16.5	8.4 ± 3.0	16.5 ± 5.9	29.0 ± 10.6	0.2 ± 0.4
100	10	38.5 ± 12.8*	7.4 ± 4.1	9.2 ± 4.1*	21.3 ± 6.8	0.6 ± 0.7
500	9	32.6 ± 7.5*	4.7 ± 1.7*	8.6 ± 2.6*	18.1 ± 6.8*	0.3 ± 0.5

Data are presented as the means ± SD. Significantly different from the untreated control group at *p < 0.05.

To clarify the mechanisms that suppress intestinal polyp formation by ITZ and TCF/LEF-, NF-κB- and NRF2-target gene expression levels in the non-polyp and polyp segments of the intestine were investigated (Fig. 4B). Real-time PCR revealed that treatment with 100 ppm ITZ for 8 weeks weakly, but significantly, suppressed *LGR5* and *COX-2* mRNA levels in the intestinal mucosa up to 70% (*p* < 0.01) and 62% (*p* < 0.005) of the untreated value, respectively. Interestingly, only *LGR5*, but not *COX-2*, mRNA expression levels were suppressed by ITZ treatment in the polyps. Although the difference was not significant, *HO-1* expression tended to increase in the mucosa and decrease in polyps.

### Abrogated cholesterol trafficking suppresses transcriptional activation of TCF/LEF and NF-κB

**Figure 5:**
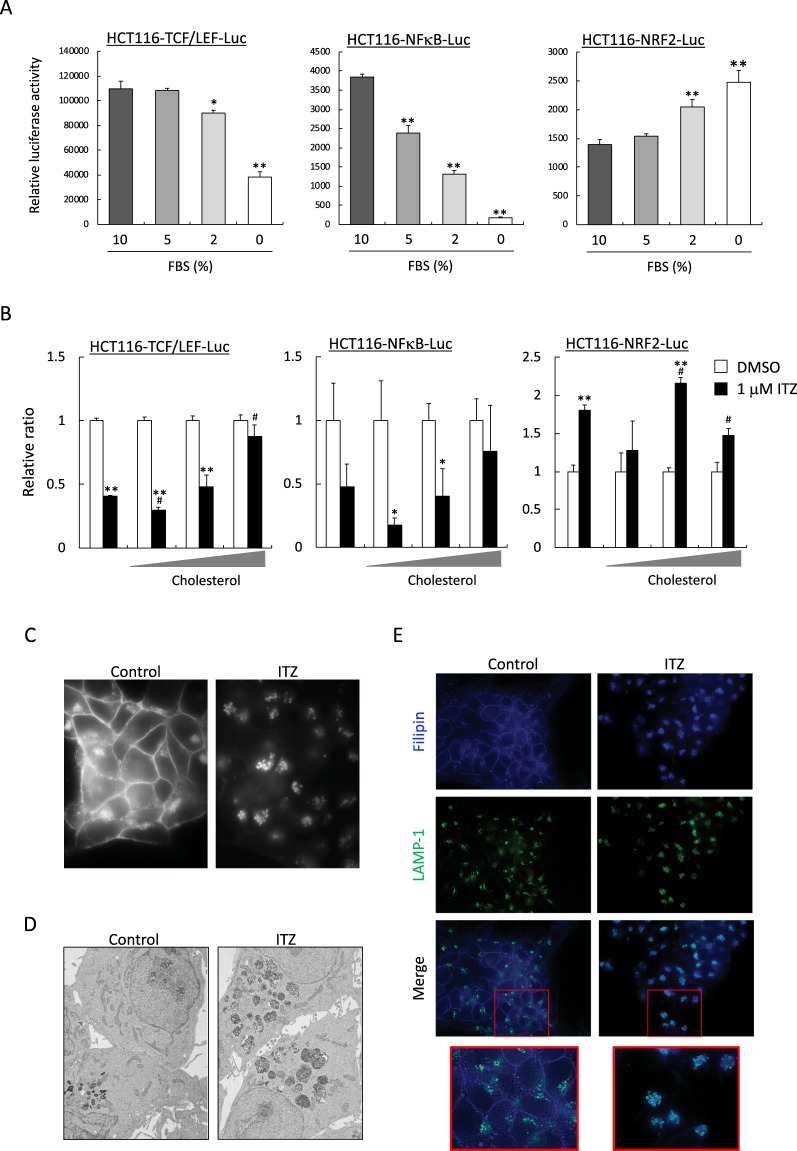
The effects of ITZ on transcriptional activities and cholesterol intracellular trafficking. (**A**) Each reporter cell line of HCT116 was seeded into 96-well plates with 10% FBS supplemented medium for 24 hours pre-incubation. Luciferase activity was measured after incubation with indicated FBS concentration for another 24 hours. The data are the mean ± SD, n = 3. *p < 0.05, **p < 0.01 *vs*. 10% FBS. (**B**) Each reporter cell line of HCT116 was seeded into 96-well plates with 10% FBS supplemented medium for 24 hours pre-incubation. Cells were then incubated for another 24 hours with 0% FBS supplemented medium with cholesterol concentrate under the presence or absence of 1 μM ITZ. Cholesterol concentrate was added at 0.1, 0.2 and 0.4% of total volume. The data are shown as the relative value, with the value of the DMSO-treated cells set as 1. The data are the mean ± SD, n = 3. *p < 0.05, **p < 0.01 *vs*. DMSO. ^#^*p* < 0.03, ^##^*p* < 0.01 *vs*. 0% FBS with 1 μM ITZ. HCT116 cells were treated with DMSO or 1 μM ITZ for 24 hours and then fixed for (**C**) filipin staining, (**D**) an electron microscope and (**E**) double staining with filipin and LAMP-1.

Cholesterol has been shown to modulates cell signaling through direct interactions with scaffold proteins^23^. While, recent paper reported that triazoles, including ITZ, inhibit cholesterol export from lysosomes by binding to Niemann-Pick C1 (NPC1)^24^. We thus wondered whether the suppression of transcriptional activations of TCF/LEF and NF-κB by ITZ was also caused by cholesterol deprivation. When FBS, as an exclusive source of cholesterol in the media, was withdrawn, transcriptional activities of TCF/LEF and NF-κB was decreased and NRF2 was increased in a dose dependent manner (Fig. 5A). Interestingly, suppressing effects of ITZ on TCF/LEF- and NF-κB-dependent transcriptional activity were canceled by cholesterol supplementation (Fig. 5B). Furthermore, ezetimibe, a cholesterol-absorption inhibitor, also suppressed TCF/LEF- and NF-κB-dependent transcriptional activities and increased NRF2-dependent transcriptional activity at 10 μM, same trend as ITZ (data not shown) suggesting that abrogation of intracellular trafficking of cholesterol affects transcriptional activities.

To assess the effect of ITZ on cholesterol intracellular trafficking, cells were stained by filipin which is cytochemical marker of cholesterol. In control cells, cholesterol was clearly distributed on the plasma membrane and perinuclear. Consistent with previous report in human umbilical vein endothelial cells, cholesterol was dramatically decrease on the plasma membrane and majority of cholesterol was within accumulated in an intracellular compartment in the cytosolic space by ITZ treatment (Fig. 5C). Further analysis using transmission electron microscopy revealed that HCT116 cells with ITZ treatment contain bloated lysosomes in the cytosolic space (Fig. 5D). These results are reminiscent of cholesterol accumulation in late endosome and lysosomes. This implication was confirmed by colocalization of cholesterol and LAMP-1, a marker of late endosome and lysosome, in ITZ treated cells (Fig. 5E). These observations suggested that ITZ inhibited cholesterol trafficking out of the lysosomes and raised the possibility that inhibition of intracellular cholesterol trafficking can lead to affect the transcriptional activations.

## Discussion

To explore novel CRC-preventive drug candidates, we established a high-throughput screening system based on luciferase activity and applied a comprehensive evaluation of TCF/LEF, NF-κB and NRF2-transcriptional activity. ITZ, one of the triazole antifungal agents, induced a decrease in the TCF/LEF and NF-κB transcriptional activity and an increase in the NRF2 transcriptional activity in this screening system. Furthermore, ITZ treatment suppressed intestinal polyp formation partly through the decreased expression of TCF/LEF and NF-κB target genes and increased expression of NRF2 target genes in Min mice. In summary, this study demonstrated the potential application of our comprehensive approach with 3 types of cell-based reporter assays in identifying novel cancer chemopreventive drugs, which may be effective in pre-clinical and clinical use.

In the first screening of the validated library (1,280 approved drugs) with our novel approach, 8 drugs (alexidine dihydrochloride, benzethonium chloride, bortezomib, docetaxel, exemestane, fenbendazole, ITZ, and pyrvinium pamoate) fulfilled the combined criteria, including decreased TCF/LEF- and NF-κB- and increased NRF2-dependent transcriptional activities. Notably, most of these 8 drugs attracted attention as novel anti-cancer agents in recent research. Although the details of the mechanism are still unclear, alexidine dihydrochloride^25^, benzethonium chloride^26^ and fenbendazole^27^ have been reported as having antitumor activities. From our results, their antitumor activities work partly through the suppressive effect on the TCF/LEF- and NF-κB-signaling pathways. Exemestane, one of the aromatase inhibitors that are well-established therapeutic drugs for breast cancer, demonstrated cancer preventive effects in the Mammary Prevention 3 (MAP.3) placebo-controlled randomized trial conducted by the National Cancer Institute of Canada^28^. In this clinical trial, exemestane significantly reduced the incidence of invasive breast cancers compared with the placebo group with no serious toxic effects. Pyrvinium pamoate, an FDA-approved major anthelmintic drug, has been reported to attenuate Wnt signaling through direct binding to and activation of CK1α^29^. Interestingly, pyrvinium pamoate was identified as a candidate to target dormant cancer cells by a glucose-deprived multicellular tumor spheroid (MCTS) screening system^30^. As MCTSs are known to closely simulate the tumor microenvironment with respect to glucose and oxygen, resulting in gene expression and phenotypic changes observed *in vivo*^31^, the candidates identified by our novel screening approach in two-dimensional culture may also be effective in a three-dimensional culture system and *in vivo*.

ITZ is a broad triazole antifungal drug developed in the 1980s^32^. ITZ is known to inhibit cytochrome p450-dependent lanosterol 14-α-demethylation in the ergosterol synthesis pathway in fungi^33^. In 2007, Chong *et al*. first reported the anti-cancer activity of ITZ through anti-angiogenic activity^34^. Treatment of ITZ alone or in combination with other anticancer drugs showed strong anticancer activities in preclinical models, including non-small cell lung cancer (NCLC), medulloblastoma and basal cell carcinoma^29,35^. Recently, positive clinical results have been reported from advanced NCLC, prostate cancer and basal cell carcinoma trials^36^. However, the effect of ITZ on intestinal carcinogenesis has not yet been elucidated even in the preclinical model. In this study, we demonstrated that ITZ administration significantly suppressed intestinal polyp formation in Min mice. All of the polyps that developed in Min mice younger than 20 weeks old were adenomas. Adenoma is premalignant lesion that can be targeted by cancer chemoprevention. Furthermore, consistent with results from *in vitro* screening, expression levels of *LGR5*, *COX-2* were significantly suppressed, and *HO-1* expression tended to be induced, in the non-polyp segments of ITZ-treated mice.

Although the precise anticancer mechanism of ITZ has remained elusive, recent studies have demonstrated that inhibition of intracellular cholesterol trafficking is one of the novel biological effects of ITZ in human endothelial cells^37,38^. In addition, recent studies suggest that intracellular cholesterol levels in evolving cancer cells might be more important than serum cholesterol^39^. Low-density lipoprotein receptor (LDLR) has been reported to regulate cholesterol uptake in cells^40^ and LDL plays an important role in growth of human colon cancer cells^41^. Interestingly, LDLR was overexpressed in polyps that highly expressed the target genes of TCF/LEF and NF-κB^42^. In addition, treatment of smooth muscle cells with LDL results in the activation of MAP kinase as well as the induction of the cell-cycle-related genes c-fos, c-myc and early growth response gene^43^. *Ldlr*-deficient mice showed a lower incidence and multiplicity of colorectal cancers compared to wild type mice in an azoxymethane (AOM) and dextran sulfate sodium (DSS)-induced carcinogenesis model, even though serum cholesterol levels were much higher in *Ldlr*-deficient mice^44^. Meanwhile, Kamisako *et al*. reported that NRF2 was involved in the induction of SREBP-1c, which is a key transcriptional regulator of genes involved in cholesterol biosynthesis and uptake^45^.

In this manuscript, we showed that itraconazole treatment induces cholesterol accumulation in the cytosolic space. As described in the Result section, it is reported that ITZ inhibits cholesterol export from lysosomes by binding to NPC1^23^. NPC1 has been identified as a gene responsible for Niemann-Pick type C (NP-C) disease. NP-C disease is characterized by lysosomal accumulation of LDL-derived cholesterol, resembling that of itraconazole treated cells. Interestingly, a reduction in Wnt signaling activity is observed in Niemann–Pick Type C disease cells^46^. Furthermore, Kuzu *et al*. reported that leelamine, an inhibitor of cholesterol egress from lysosomes, reduced cholesterol levels in all membrane-bound organelles in cancer cells and suppressed NF-κB activation *via* the Akt signaling pathway^47^.

Furthermore, we have shown the effects of ezetimibe on transcriptional activities of TCF/LEF, NF-κB and NRF2, as appears in Fig. S2. Ezetimibe is a selective cholesterol absorption inhibitor, which potently inhibits Niemann-Pick C1 Like 1 (NPC1L1). NPC1L1 is a multi-transmembrane protein playing a crucial role in formation and endocytosis of NPC1L1-flotillin-cholesterol membrane microdomains, which is an early step in cholesterol uptake into intestinal epithelial cells. As shown in Fig. S2, ezetimibe treatment suppressed TCF/LEF and NF-κB transcriptional activities, and induced NRF2 transcriptional activity as well as itraconazole did. Although additional studies are needed to elucidate the mechanisms in detail, these results suggest that abrogation of cholesterol trafficking may affect signaling pathways that may be involved in the early stage of colon carcinogenesis (Wnt, NF-κB, NRF2).

In the recent study, we have revealed that ITZ and ezetimibe, inhibitors of cholesterol intake, can suppress TCF/LEF- and NF-κB-dependent transcriptional activities and induce NRF2-dependent transcriptional activities partly through the inhibition of intracellular cholesterol trafficking, resulting in cholesterol depletion. Taken together, cholesterol depletion within the cells by inhibition of intake and intracellular trafficking may be a novel target for colon cancer prevention.

In this study, we have established a novel screening approach and identified 2 candidates with high potential as CRC-preventive drugs. Although further studies should be performed to elucidate the molecular mechanism in detail, compounds that are hits from our screening system could be good candidates for colon cancer-preventive drugs in clinical use.

## Materials and Methods

### Cell cultures and chemicals

The human colon cancer cell lines HCT116 and DLD-1 were purchased from American Type Culture Collection (Manassas, VA, USA). The cells were maintained in DMEM supplemented with 10% heat-inactivated fetal bovine serum (FBS; HyClone Laboratories Inc., Logan, UT, USA) and antibiotics (100 μg/mL streptomycin and 100 U/mL penicillin) at 37 °C in 5% CO_2_. The “validated library”, consisting of 1280 approved drugs, was obtained from the Open Innovation Center for Drug Discovery (The University of Tokyo, Japan). TNF-α, tBHQ and iCRT14 were purchased from Miltenyi Biotec (Bergisch Gladbach, Germany), TCI (Tokyo, Japan) and Sigma-Aldrich (St. Louis, MO, USA), respectively. 250X Cholesterol Lipid Concentrate were purchased from Gibco Japan Ltd. (Tokyo, Japan).

### Establishment of stable transfectants with TCF/LEF-, NF-κB-, NRF2-reporter vectors

To establish stable transfectants with the TCF/LEF-, NF-κB-, NRF2-reporter vectors, the colon cancer cell lines HCT116 and DLD-1 were seeded in 100-mm dishes (3.0 × 10^7^ cells/dish). After 24 hours of incubation, the cells were transfected with 15 µg of either pGL4.49 [*luc2P*/TCF-LEF-RE/Hygro], pGL4.32 [*luc2P*/NF-κB-RE/Hygro] or pGL4.37 [*luc2P*/ARE/Hygro] (Promega, Madison, WI, USA) plasmid using Polyethylenimine “MAX” Transfection Reagent (Polysciences Inc., Warrington, PA, USA) for HCT116 cells and X-tremeGENE HP DNA transfection reagent (Roche, Mannheim, Germany) for DLD-1 cells according to the instructions provided by the manufacturer. After 6 hours of transfection, cells were cultured with fresh media for 48 hours and then passaged in medium containing hygromycin at 0.1 mg/ml for HCT116 cells and 0.25 mg/ml for DLD-1 cells. Hygromycin selection was conducted for at least 2 weeks followed by colony selection. To validate the response against the respective inducer or inhibitor, each reporter cell line was stimulated by TNF-α (1.25, 2.5, 5, 10, 20, 40 ng/ml), tBHQ (1.25, 2.5, 5, 10, 20, 40 μM), or Wnt3a conditioned medium (CM) and iCRT14 (5, 10 μM) in HCT116 cells or iCRT14 (20, 40 μM) in DLD-1 cells. Mouse fibroblast L cells expressing Wnt3a were purchased from ATCC (Manassas, VA, USA) and maintained in DMEM. Wnt3a conditioned medium was prepared according to the ATCC protocol.

### Screening of validated library

For the first screening, HCT116 and DLD-1 cell lines that were stably transfected with the TCF/LEF-reporter vector were seeded into black 96-well half-area microplates (Corning) and cell lines stably transfected with the NF-κB- and NRF2-reporter vectors were seeded into white 96-well half-area microplates (Corning) at a density of 1 × 10^4^ cells per well in 50 μl of media. After 24 hours pre-incubation, each test compound was diluted in DMSO or DMSO alone and added into the culture plate at 1 μM as a 1% final concentration for another 24 hours. Then, Glo Lysis Buffer (Promega, Madison, WI, USA) followed by Bright-Glo reagent was dispensed into each well, and the luminescence signals for the individual wells were measured using a TECAN plate reader. The relative luciferase activity was normalized to the DMSO control as 1.0. For the second screening, the effects of the primary hits on the luciferase activities of each were re-evaluated at 0.01, 0.1 and 1 μM. The luciferase activity was normalized to protein concentration, which was determined using a Pierce 660-nm protein assay (Thermo Scientific, Rockford, IL, USA) according to the manufacturer’s instructions with BSA as a standard. Each sample was measured in triplicate.

### Animals

Female C57BL/6-*Apc*^Min/+^ mice (Min mice) were purchased from The Jackson Laboratory (Bar Harbor, ME, USA). The mice were housed in plastic cages with sterilized softwood chips as bedding in a barrier-sustained animal room at 24 ± 2 °C and 55% humidity on a 12-hour light/dark cycle. Itraconazole (ITZ) was well mixed at a concentration of 100 or 500 ppm in an AIN-76A powdered basal diet (CLEA Japan, Tokyo, Japan).

### Protocol for animal experiments

Ten female Min mice at 5 weeks of age were given 100 or 500 ppm ITZ for 8 weeks. The animals in the same cage were all in the same treatment group. Food and water were available *ad libitum*. The animals were observed daily for clinical signs. Body weights and food consumption were measured weekly. The intestinal tract was removed and separated into the small intestine, cecum and colon. The small intestine was divided into proximal segments (4 cm in length), and then the remaining part was divided into a proximal (middle) and distal segment. Polyps in the proximal segments were counted, and all polyps in the proximal segment were excised under a stereoscopic microscope. The remaining intestinal mucosa (non-polyp portion) was removed by scraping, and both segments were stored at −80 °C for further analysis. Other segments were opened longitudinally and fixed between sheets of filter paper in 10% buffered formalin. The numbers, sizes and distributions of polyps in the intestine were assessed with a stereoscopic microscope. The experiments were performed according to the “Guidelines for Animal Experiments in the National Cancer Center” and approved by the Institutional Ethics Review Committee for Animal Experimentation at the National Cancer Center.

### Quantitative real-time polymerase chain reaction (PCR) analysis

Total RNA was isolated using RNAiso Plus (TaKaRa, Shiga, Japan) and 1-μg aliquots in a final volume of 20 μL were used for synthesis of cDNA using a High Capacity cDNA Reverse Transcription kit (Applied Biosystems, Foster City CA, USA). Real-time PCR was performed using the CFX96/384 PCR Detection System (BIO RAD, Tokyo, Japan) and Fast Start Universal SYBR Green Mix (Roche Diagnostics, Mannheim, Germany), according to the manufacturer’s instructions. The following primers were used to evaluate the levels of human mRNA: LGR5 (5′-CTC CCA GGT CTG GTG TGT TG and 5′-GAG GTC TAG GTA GGA GGT GAA); COX-2 (5′-GAT ACT CAG GCA GAG ATG ATC TAC CC and 5′-AGA CCA GGC ACC AGA CCA AAG A); HO-1 (5′-CCA GGC AGA GAA TGC TGA GT and 5′-GTA GAC AGG GGC GAA GAC TG); and glyceraldehyde-3-phosphate dehydrogenase (GAPDH) (5′-CCA CCC ATG GCA AAT TCC and 5′-TGG GAT TTC CAT TGA CAA). The primer sequences for mouse samples were as follows: LGR5 (5′- TGC CCC GTG GCT TTC TTA TC and 5′-TTT CCC AGG CTG CCC ATA TC); COX-2 (5′-GTG CCA ATT GCT GTA CAA GC and 5′-TAC AGC TCA GTT GAA CGC CT); and GAPDH (5′-TGT CAG CAA TGC ATC CTG CA and 5′-TTA CTC CTT GGA GGC CAT GT). To assess the specificity of each primer set, the melting curves of amplicons generated from the PCR reaction were analyzed.

### Filipin and Immuno-staining

Cells were fixed with 4% paraformaldehyde in PBS at room temperature for 15 minutes. Cells were then permeabilized with 0.1% Triton X-100 in PBS at room temperature for 5 min and blocked with 1% goat serum in PBS (blocking buffer) for 30 min. Cells were then incubated with LAMP-1 antibody (Cell signaling Technologies, #9091) in blocking buffer at room temperature for 1 hour, followed by incubation with secondary antibody (Invitrogen, #A-11008) in blocking buffer at room temperature for 30 min. Cells were then stained with 50 μg/ml filipin in PBS at room temperature for 2 hours. After filipin staining, cells were washed with PBS three times and mounted. Images were captured using an all-in-one fluorescence microscope (BZX-710; Keyence).

### Electron microscope

For transmission electron microscopy, cells were fixed with 2% paraformaldehyde and 2% glutaraldehyde (GA) for 30 min at 4 °C, followed by fixation with 2% GA at 4 °C overnight. Specimens were fixed with 2% osmium tetroxide for an additional 1 hour at 4 °C, dehydrated in alcohol and embedded in resin (Nisshin EM Co., Tokyo, Japan). Ultra-thin sections were stained with 2% uranyl acetate and Lead stain solution (Sigma-Aldrich Co., Tokyo, Japan) and photographed using JEM-1400Plus transmission electron microscope (JEOL Ltd., Tokyo, Japan).

### Statistical analyses

The results are expressed as the mean ± SD, with statistical analysis using Student’s *t*-test, except for polyp number in Min mice (One-way ANOVA). Differences were considered significant at *p* < 0.05.

## Supplementary information

Supplementary Information for Novel screening system revealed that intracellular cholesterol trafficking can be a good target for colon cancer prevention
