# Supplementary material for: Novel screening system revealed that intracellular cholesterol trafficking can be a good target for colon cancer prevention
Source: Sci Rep. 2019 Apr 17;9:6192. doi: 10.1038/s41598-019-42363-y (PMC6470178; doi:10.1038/s41598-019-42363-y)
Supplement: Supplementary file 1 — Supplementary Information for Novel screening system revealed that intracellular cholesterol trafficking can be a good target for colon cancer prevention [file 41598_2019_42363_MOESM1_ESM.pdf]

Supplementary Information for

Novel screening system revealed that intracellular cholesterol trafficking can be a good target for colon cancer prevention

Shingo Miyamoto[1,#], Takumi Narita[1], Masami Komiya[1], Gen Fujii[2], Takahiro Hamoya[1], Ruri Nakanishi[1], Shuya Tamura[1], Yurie Kurokawa[1], Maiko Takahashi[1], Michihiro Mutoh[1,*]

[1]Division of Prevention, Center for Public Health Sciences, National Cancer Center [2]Central Radioisotope Division, National Cancer Center Research Institute [#]Present affiliation: Department of Cancer Cell Research, Sasaki Institute, Sasaki Foundation

*To whom correspondence should be addressed. Michihiro Mutoh, Division of Prevention, Center for Public Health Sciences, National Cancer Center, 5-1-1 Tsukiji, Chuo-ku, Tokyo 104-0045, Japan. Tel: +8133542251, Fax: +8133543905, E-mail: mimutoh@ncc.go.jp

Supplementary Information includes:

Supplementary Figure S1

Supplementary Figure S2

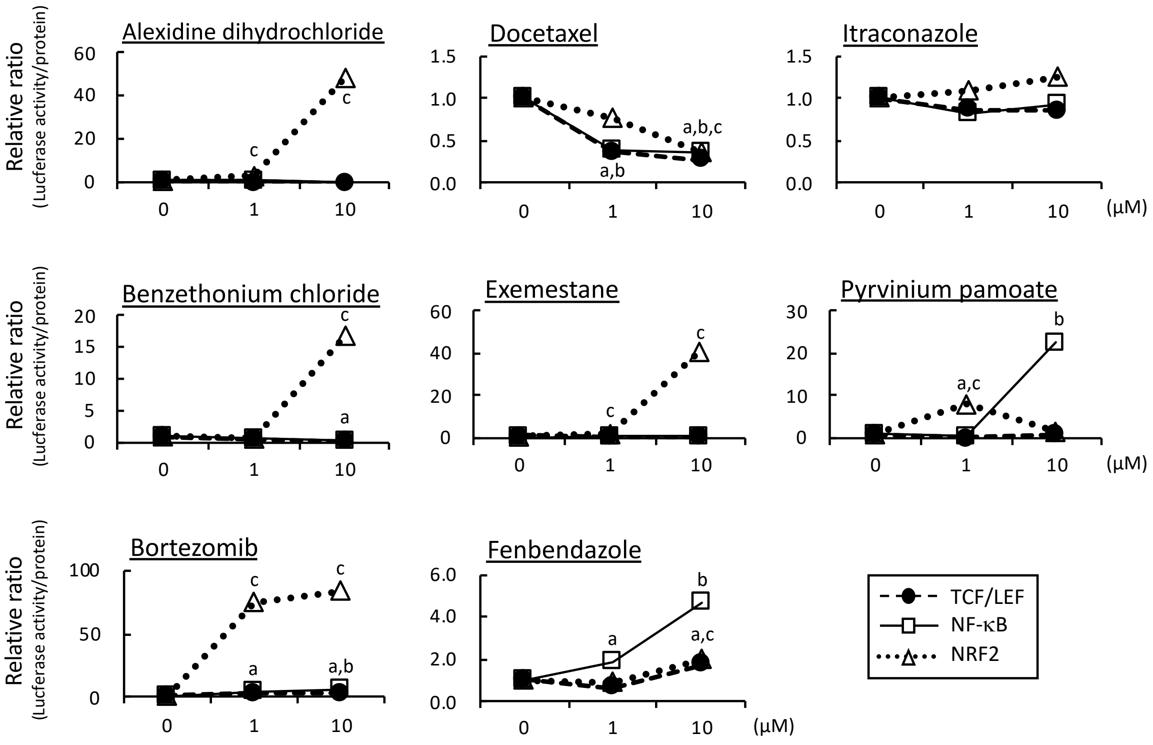

Supplementary Figure 1. Secondary screening of primary hits with the data of a strong candidate, itraconazole. The dose-dependent effects of the 8 compounds that were primary hits by first screening were evaluated in HT29 cells. HT29 cells were stably transfected with the TCF/LEF-, NF-κB- and NRF2-reporter vectors, and the response validated against the respective inducers or inhibitor, as well as HCT116 cells. The effects of each compound on luciferase activity were evaluated and normalized by protein concentration in the same way as the protocol for HCT116 cells. The data are indicated as the mean, n = 3. [a] $p < 0.05$ *vs*. control in TCF/LEF. [b] $p < 0.05$ *vs*. control in NF-κB. [c] $p < 0.05$ *vs*. control in NRF2.

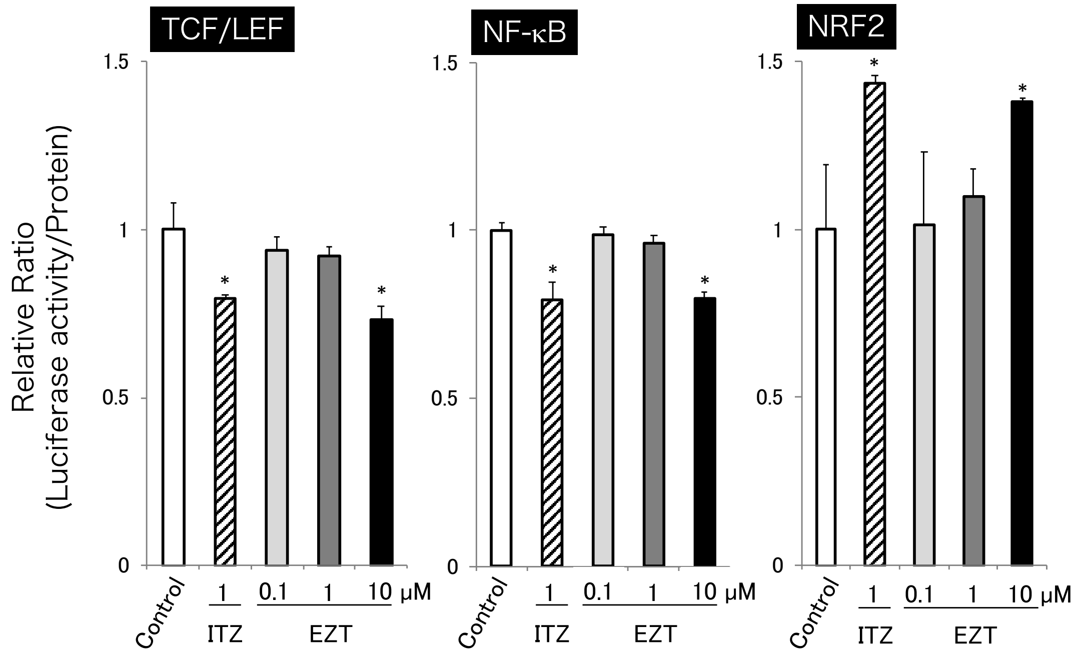

Supplementary Figure 2. Ezetimibe behaves in a similar way to itraconazole in its action on the activities of TCF/LEF-, NF-κB- and NRF2-transcription.

The effects of ezetimibe (EZT) were evaluated and normalized by protein concentration. HCT116 reporter cell lines were seeded into 96-well half-area microplates at a density of $1x10^4$ cells per well in 50 μl of media. After 24 hours pre-incubation, ezetimibe diluted in DMSO or DMSO alone was added to the culture plate at 0.1, 1, 10 μM as a 1% final concentration for another 24 hours. Itraconazole (ITZ) was added to the culture plate at 1 μM. Relative luciferase activity was evaluated 24 hours after adding EZT and ITZ to HCT116 reporter cell lines. The relative luciferase activity was normalized by protein concentration and to the DMSO control as 1.0. The data are indicated as the mean, n = 3. $^{*}p < 0.05$ *vs.* control
